# Traumatic Separation of a Bipartite Patella With Concurrent Quadriceps Tendon and Retinacular Injury: A Report of a Rare Case

**DOI:** 10.7759/cureus.96750

**Published:** 2025-11-13

**Authors:** Emmanouil Kroustalakis, Michail Kroustalakis, Nikolaos Ritzakis, Panayiotis Ioannou, Konstantinos Zampetakis

**Affiliations:** 1 Department of Orthopaedics and Traumatology, "Venizeleio" General Hospital of Heraklion, Heraklion, GRC; 2 Department of Orthopaedics and Traumatology, University General Hospital of Patras, Patras, GRC

**Keywords:** bipartite patella, knee trauma, quadriceps tendon injury, retinaculum, screw fixation, separation

## Abstract

Bipartite patella is a developmental variant that typically remains unrecognized but may rarely comprise an incidental finding following trauma. Traumatic separation of a bipartite patella is a rare clinical entity that can mimic a patellar fracture. The concurrent presence of complete quadriceps tendon rupture and retinacular injury is extremely uncommon. We describe the case of a 61-year-old male who presented after a ground-level fall with anterior knee pain, palpable suprapatellar gap, joint effusion, and inability to actively extend the knee. Radiographs revealed separation of the patella with smooth fragment margins, suggestive of a bipartite patella rather than a true fracture. No supplementary imaging was performed. Intraoperative findings confirmed the diagnosis of bipartite patella with accompanying complete quadriceps tendon rupture and bilateral retinacular disruption. Surgical management involved the preservation and fixation of the patellar fragment with a compression screw, combined with restoration of the extensor mechanism employing quadriceps tendon repair with bony anchors. Postoperative follow-up demonstrated a stable fragment position and favourable functional outcomes with restoration of knee stability and near-complete range of motion. This case highlights screw fixation of the patellar fragment as a viable therapeutic strategy in the setting of bipartite patella separation with concurrent complete quadriceps tendon rupture. By demonstrating a successful outcome with this rarely used treatment modality, this case expands the limited knowledge base available to guide clinicians in managing similar cases.

## Introduction

Traumatic separation of a bipartite patella with coexisting injury of the quadriceps tendon and retinacula represents an exceptionally rare injury pattern [1]. Bipartite patella constitutes a developmental variant, with a reported prevalence of 0.2-6% in adults, which occurs due to fusion failure of patellar secondary ossification centers [2,3]. This condition becomes symptomatic in only 1-2% of cases, and usually it is an incidental finding, mainly following a traumatic event [1,4].

To the best of our knowledge, the first case of bipartite patella separation was described in 1989 by Carter [5]. Since then, only a small number of isolated cases have been reported in the literature, and most often, the injury mechanism involves a forced quadriceps contraction felt as sudden anterior knee pain with an accompanying audible pop [6]. In a few cases, associated quadriceps tendon rupture is reported, and even fewer have demonstrated concurrent injury to the retinaculum. This injury combination raises an important clinical challenge, as the separated fragment management can affect the extensor mechanism integrity [7].

Treatment options for traumatic separation range from non-operative to operative management, and the choice is guided by fragment size, articular cartilage involvement, soft tissue attachment, and the surgeon’s preference. When surgery is indicated, the most common technique is fragment excision with reported favorable outcomes [6,8]. However, in only a very limited number of published cases, open reduction with fragment fixation using tension band or screws is the treatment of choice [1].

We present a rare case of traumatic separation of a bipartite patella with concurrent quadriceps tendon and retinacular injury, successfully treated with screw fixation of the accessory ossicle along with soft-tissue repair. This case underscores diagnostic considerations and therapeutic rationale in this rare but clinically significant injury constellation. This report was prepared according to CAse REport (CARE) guidelines, and informed consent was obtained for publication.

## Case presentation

A 61-year-old male patient presented to the Emergency Department due to a left knee injury after sustaining a ground-level fall, landing with direct impact onto the affected knee. His past medical history consisted of hypertension, dyslipidaemia, tachycardia, and sleep apnoea, while his family history was unremarkable. The patient’s main complaint was left knee pain with concurrent inability to bear weight. During clinical examination, joint effusion was present along with localised tenderness and a palpable defect over the superior pole of the patella. In addition, the inability to actively extend the left knee was noted, suggesting disruption of the extensor mechanism.

**Figure 1:**
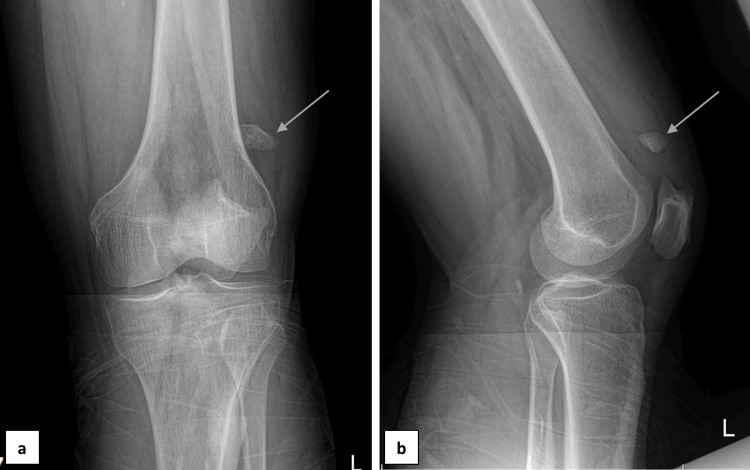
X-ray images of the left knee Anteroposterior (a) and lateral (b) X-ray views of the left knee indicating separation of Saupe Type III bipartite patella. The arrows indicate the separated anterosuperior patellar fragment.

The anteroposterior (AP) and lateral radiographs (Figure 1) of the injured knee depicted a linear discontinuity of the superolateral pole of the left patella with a clear gap between the fragments. The rounded borders of the patellar fragments raised the suspicion of separation of Type III bipartite patella (superior/lateral) according to Saupe’s classification [9]. The contralateral side was not examined at that moment since no discomfort was present. A long leg splint was applied for support and comfort. As no further imaging was obtained, a decision was made to proceed with admission and surgical intervention based on clinical presentation and plain radiographs.

**Figure 2:**
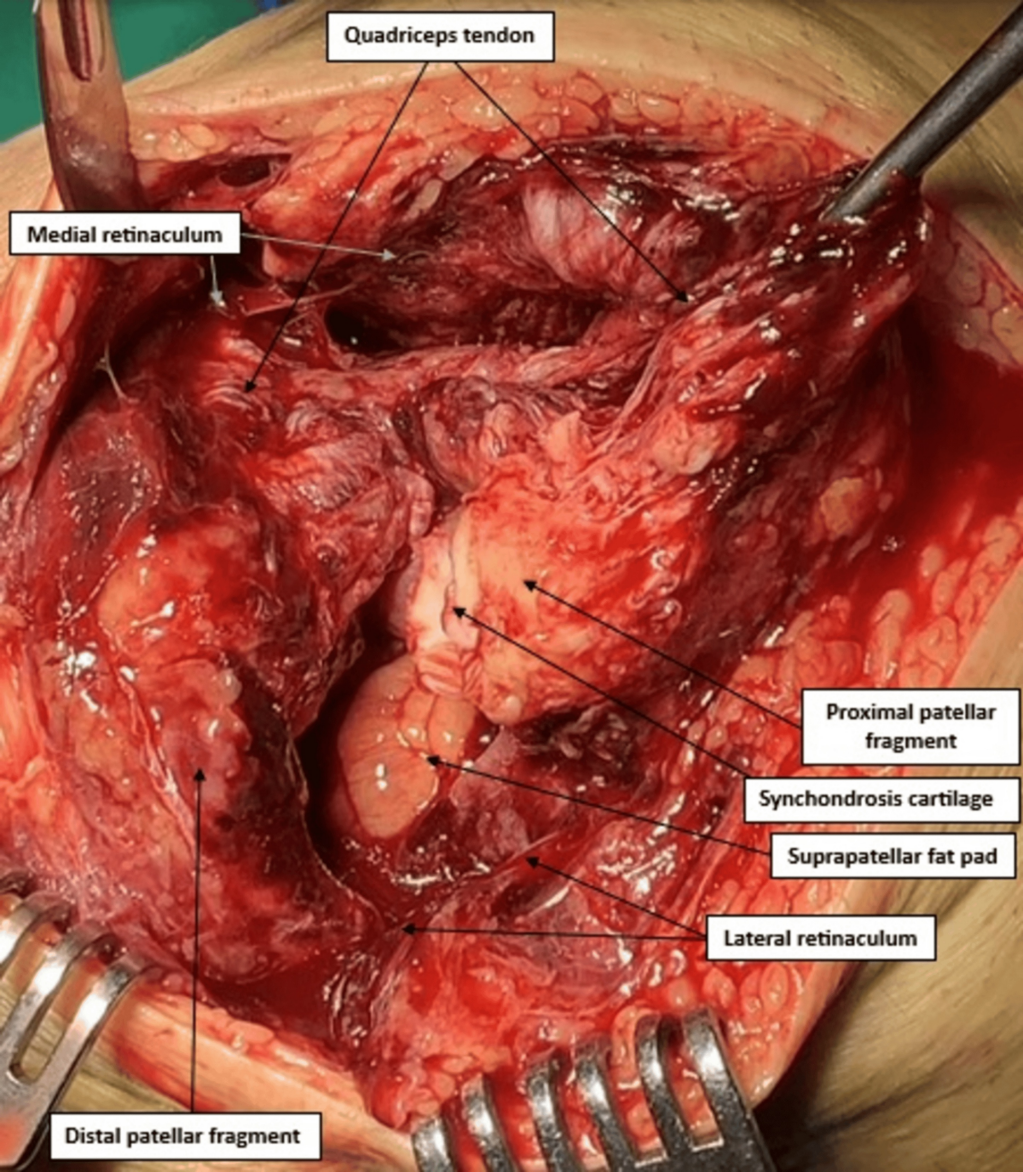
Intra-operative findings of bipartite patella separation Relevant structures are labeled by arrows.

The surgery was performed the next day under regional anaesthesia. A midline longitudinal incision was performed. After sharp dissection and full-thickness flaps development, the two bony fragments of the patella were identified. The proximal, smaller one was retracted superiorly by the quadriceps tendon, and the distal fragment was held in place by the patellar tendon. The diagnosis of bipartite patella was confirmed intraoperatively, and the connecting cartilage between the fragments was identified and attached to the proximal part. Moreover, the remaining extensor mechanism was disrupted, as the quadriceps tendon was ruptured with extension to the medial and lateral retinacula (Figure 2).

**Figure 3:**
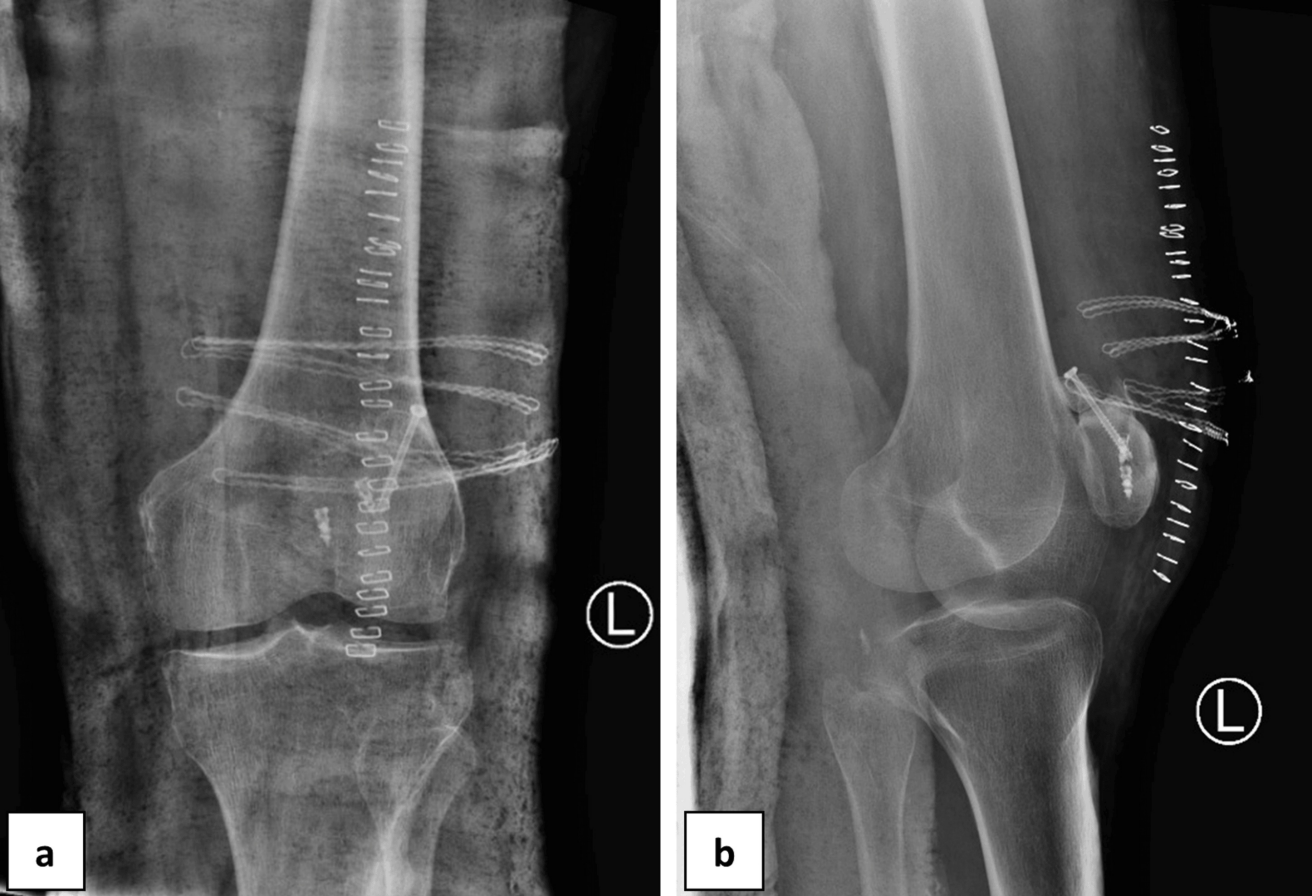
Poste-operative X-ray images Post-operative anteroposterior (a) and lateral (b) X-ray views of the left knee indicating fixation of bipartite patella separation using a canulated partially threaded screw. The quadriceps tendon was reattached using metallic bony anchors.

Debridement and reduction of the bony fragments were carried out, and fixation was performed using a cannulated partially threaded screw. The quadriceps tendon rupture was addressed by employing the modified Kessler technique and was fixated on the superior pole of the inferior patella fragment with the aid of two metallic bony anchors. During the whole procedure, fluoroscopic evaluation of the fixation was conducted with a favourable result. A long leg splint was implemented with the knee in full extension. The patient was discharged the following day. The postoperative radiographs of the patient are illustrated in Figure 3.

**Figure 4:**
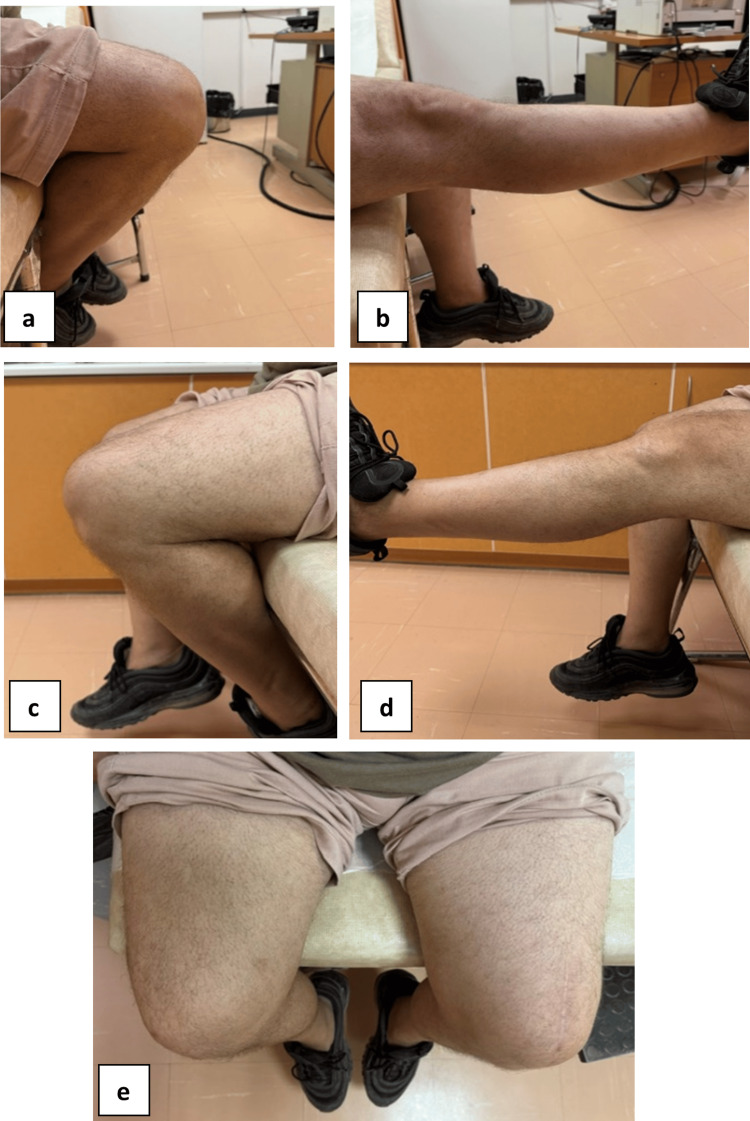
Follow-up clinical images of the patient Follow-up clinical images illustrating knee range of motion: (a) right knee in full flexion, (b) right knee in full extension, (c) left knee in extension with a 5° lag, (d) left knee in full extension, and (e) superior view of both knees in flexion.

**Figure 5:**
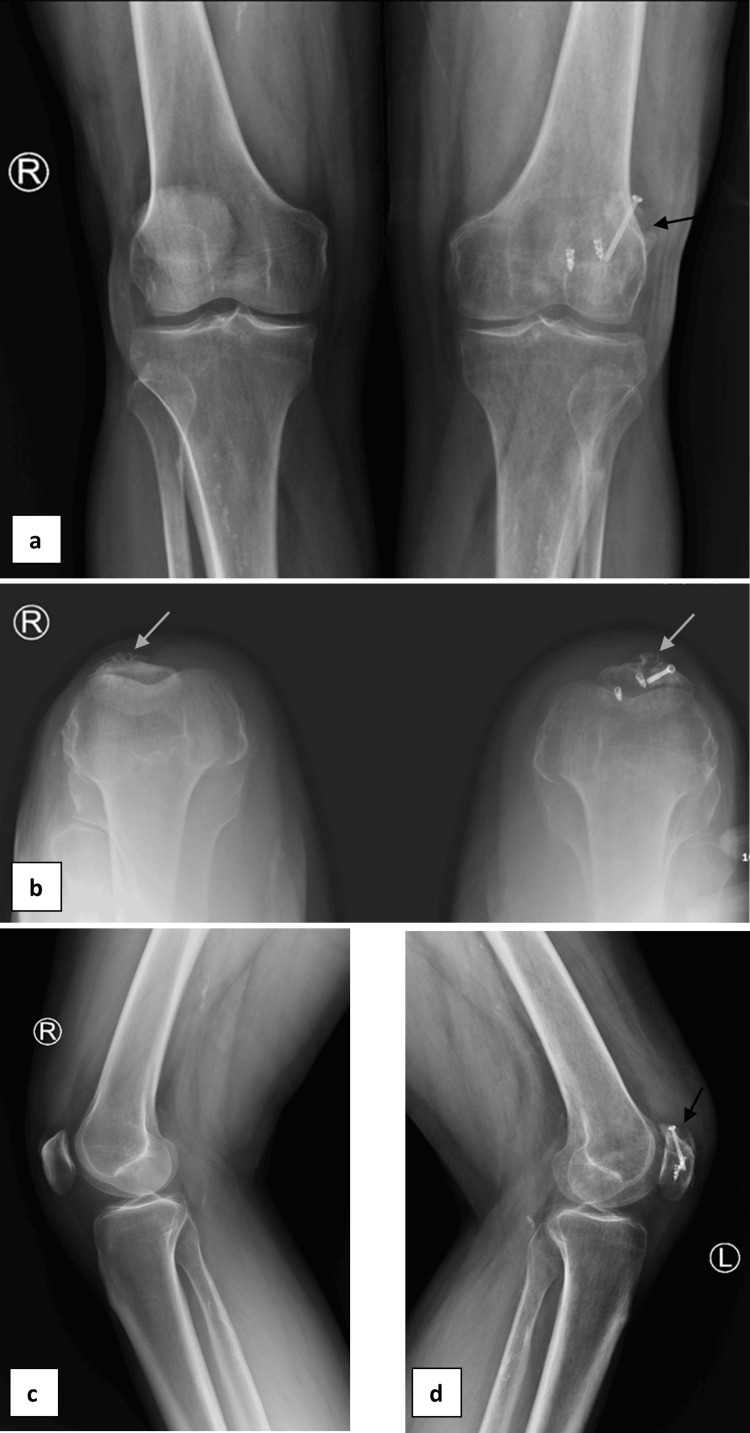
Follow-up radiographs of both knees (a) Standing AP view, (b) skyline view, showing osteophytes along the anterior cortex of the patella, more pronounced on the left side (grey arrows), (c) right lateral view, and (d) left lateral view. The black arrows indicate the stable position of the fixation.

Partial weight-bearing was advised for the following six weeks, with immobilization of the knee joint in extension. Then, a knee brace was applied, allowing gradual increasing flexion of the knee by 10 degrees every week. At six months following surgery, the patient reported that he was doing well with no pain. There was no residual edema, and the active range of motion of both knees was almost identical, with 5 degrees of extension deficit on the affected side (Figure 4). Tegner Lysholm Knee score was at 82/100, while Kujala score regarding the anterior knee pain was 86/100. Finally, bilateral knee x-rays were obtained at follow-up, including weight-bearing views (Figure 5). No evidence of a bipartite patella was observed in the contralateral knee. The fixation of the bipartite patella separation in the affected knee appeared in a stable position. No callus formation or direct healing was observed, consistent with the presence of interposed fibrocartilage at the synchondrosis.

## Discussion

The patella is a sesamoid bone and an indispensable part of the knee extensor mechanism, surrounded by the quadriceps tendon superiorly and the patellar tendon inferiorly. In the early stages of life, it consists of cartilage and begins to ossify between the ages of two and six years [10]. The ossification of the patella typically takes place from a single primary ossification center. However, in approximately 23% of cases, multiple ossification centers are present, which usually coalesce during development to form a single patellar bone [11]. Although rare, failure of these ossification centers to properly fuse can lead to different segmental variations, like bipartite, tripartite, or multipartite patella. Even though the bipartite patella was first described in 1883 by Gruber, while performing an autopsy on a 21-year-old farmer, classification of these variations was proposed long after by Saupe in 1943 [1]. In his categorization, three different types of bipartite patella were introduced, related to the location of the accessory fragment. In detail, in type I the fragment is located on the inferior pole of the patella, in type II on the lateral margin, and in type III in the superior/lateral border [9]. Due to the limitation of Saupe’s categorization to describe the multipartite patella variations, Oohashi et al. presented a new classification in 2010 applicable to all types of segmental variations of the patella [12].

Bipartite patellae are usually asymptomatic and become an incidental discovery following a traumatic injury. Separation of the bipartite patella is an extremely rare injury, and bibliographic reports associated with a complete quadriceps tendon rupture are even more scarce [7]. Woods et al. published a case in 2007 regarding a 44-year-old male with bipartite patella who sustained a complete quadriceps rupture treated by excision of the patellar fragment and quadriceps tendon repair [13]. Mohammad et al. in 2014 reported a complete rupture of the quadriceps tendon with retinacular injury and bipartite patella separation in a 32-year-old patient. This case was addressed by screw fixation of the patellar fragment and repair of the tendon tear utilizing bony anchors [14]. Later, in 2020, Naikoti et al. presented another case of bipartite patella with complete quadriceps tendon rupture in a 32-year-old patient, managed by internal fixation of the patella using tension band followed by quadriceps tendon repair [1]. More recently, in 2021, Miles et al. demonstrated a case of a 48-year-old man with a bilateral multipartite patellar avulsion associated with a unilateral quadriceps tendon rupture. In this case, excision of the patella fragment was performed, and the quadriceps tendon was reattached to the patella [2]. To our knowledge, following the case of Mohammad et al., our case represents only the second report of bipartite patella separation with complete quadriceps tendon disruption and retinacular injury, managed with internal fixation of the patellar fragment using a compression screw.

The present case highlights a rare injury pattern and demonstrates successful management with favorable functional outcomes, offering useful guidance to the limited existing literature. Intraoperative findings confirmed the presence of a fibrocartilaginous synchondrosis. A labeled intraoperative figure (Figure 2) is included to demonstrate anatomical relationships, and our follow-up established both clinical recovery and radiographic stability. Certain limitations should also be acknowledged. Further imaging, including ultrasound, MRI or CT, was not obtained preoperatively, which might have added diagnostic precision. Moreover, as with any single case, the observations cannot be generalized, and considering the relatively short follow-up duration, the assessment of long-term outcomes is restricted.

Our decision to proceed with operative management in this patient was based on clinical and radiographic findings, such as disruption of the extensor mechanism, a palpable gap in the suprapatellar region, inability to bear weight, and the presence of rounded fragment displacement in the X-ray films. Intraoperative findings supported the diagnosis of bipartite patella separation, as a fibrocartilaginous interface was present, reinforcing the preoperative assessment. Preservation of the patellar fragment and fixation using a compression screw were used to securely restore the anatomy of the patella and the extensor mechanism, leading to a favorable functional outcome. Compared with the very limited documented cases, this case report favors the rationale that open reduction and internal fixation using a screw is a reliable choice in selected cases, when the patellar fragment is large enough and accompanying soft tissue injury is present. It highlights the value of clinical and radiographic diagnosis and advocates that timely surgical treatment can yield excellent results in this rare injury pattern.

## Conclusions

Traumatic bipartite patella separation with concurrent complete quadriceps tendon and retinacular injury is an extremely rare clinical entity that may resemble a patellar fracture. A cautious evaluation of plain X-rays and intraoperative findings was important for establishing the diagnosis of this case. Screw fixation of the patellar fragment is not a routinely used therapeutic approach, but it can restore the continuity of the knee extensor mechanism and lead to a favorable functional outcome. This case report highlights the necessity for including bipartite patella separation in the differential diagnosis of anterior knee trauma and supports surgical fixation with a screw as an effective treatment modality for this scarcely reported injury.
